# Translation and validation of diabetes self-management profile (DSMP) into Brazilian Portuguese language: first instrument to assess type 1 diabetes self-management in a pediatric population

**DOI:** 10.1186/s13098-017-0250-0

**Published:** 2017-07-11

**Authors:** Caroline Gouveia Buff Passone, Lygia Spassapan Oliveira Esteves, Roberta Dias Savoldelli, Michael A. Harris, Durval Damiani, Thais Della Manna

**Affiliations:** 1 Pediatric Endocrinology Unit, Instituto da Criança do Hospital das Clínicas da Faculdade de Medicina da Universidade de São Paulo, Rua Joaquim Távora, 550, ap123a, São Paulo, Brazil; 2 Pediatrics & Anesthesiology, Oregon Health & Science University, Portland, OR USA

## Abstract

**Objective:**

To translate and validate the instrument Diabetes Self-Management Profile (DSMP)—Conventional and Flexible Regimens into Brazilian Portuguese language in order to evaluate the quality of diabetes self-management in children and adolescents with type 1 diabetes and their caregivers.

**Methods:**

DSMP was submitted to forward and back translation method and validated in a group of type 1 diabetes youths between 6 and 18 years (n = 102), and their families. Analysis of DSMP internal consistency, intra and interobserver reliability and concurrent correlation with HbA1c were done.

**Results:**

DSMP total scores demonstrated adequate internal consistency (Cronbach’s α = 0.79), 3-month test–retest reliability (ρ = 0.53; p < 0.001), inter-interviewer agreement (ρ = 0.55; p < 0.001). DSMP total score was significantly correlated to HbA1c (ρ = −0.54, p < 0.001).

**Conclusion:**

DSMP-translated version is a reliable and valid tool to assess diabetes self-management.

## Background

Type 1 diabetes (DM1) management requires that patient and family adopt a complex treatment regimen that is focused on continuing education by health care professionals. The partnership between an informed and proactive multidisciplinary team and motivated patients who are active in their self-management is essential for an effective control of the disease.

Many instruments were developed to analyze the quality of self-management in diabetes but none have already been validated for young children in Brazilian Portuguese language. Given the current growing number of DM1 cases in childhood in Brazil, estimated in 30,900 children under 15 years old, with an expectation of 5000 new cases per year [1, 2], there is still a lack of a diabetes self-management assessment tool specific to this population in order to identify their immediate education needs.

After evaluating several tools, we chose to translate DSMP as it contemplates a more comprehensive age range, has the highest correlation with HbA1c and with quality of life, and also includes evaluation of patients in flexible regimen [3].

The original DSMP validation was obtained in a study with 105 patients showing adequate internal consistency for DSMP-Conventional Regimen score [4]. After that, DSMP-Flexible Regimen was validated in 2005 [5], 2010 [6], and translated into Spanish [7] and Brazilian Portuguese language in adults and adolescents [8].

## Objective

The aims of this study were to translate the instruments DSMP-Conventional and Flexible Regimens into Brazilian Portuguese language and to validate them in a local population of children and adolescents with DM1, and their caregivers, in order to evaluate the quality of diabetes self-management.

## Methods

DSMP is a semi-structured interview tool that evaluates diabetes self-management in the last three months, comprising 25 questions divided into five domains or subscales: exercise (frequency and insulin adjustment), hypoglycemia (management of hypoglycemia and use of diabetes identification accessories), eating (quantity, quality, insulin adjustment to food), glucose monitoring (frequency of blood glucose monitoring and use of ketonuria/ketonemia strips), insulin (regularity, missing of insulin doses, adjustment).

Initially, DSMP was translated into Portuguese language according to an interactive process of forward and back translation [9]. The translated version was applied to five patients followed in our outpatient clinic and their caregivers, and items that were misunderstood due to translation were identified and amended.

The final DSMP version was administered to patients with DM1 aged 6–18 years old who were being treated regularly in the Diabetes Outpatient Clinic of Instituto da Criança (HCFMUSP) between 2012 and 2013, and to their caregivers. Patients with concurrent medical conditions like developmental disability, psychiatric disorder or with insulin dose less than 0.5 U/kg/day were excluded from this study.

The study population characterization included gender, age, diabetes duration, family income, main caregiver and HbA1c levels at the time of the interview or the mean level of the two tests carried out over a period of 3 months before the interview. The analytical method was Biorad HPLC Variant II. Values were analyzed quantitatively.

The same researcher interviewed both the youth and caregiver using one of the two forms: the DSMP Conventional Regimen (patients with regular or rapid-acting insulin in fixed doses) and the DSMP Flexible Regimen (participants whose prescribed regimen included carbohydrate counting). Children under 11 answered the interview questions along with their caregivers, while older patients did it by themselves.

Each interview took approximately 20 min and it was repeated twice by the same researcher within a 3-month period; it was recorded and analyzed by an additional researcher to ensure intra and inter-rater agreement, respectively.

Cronbach’s α [11] was calculated to check for internal consistency of the whole DSMP interview and of each the five subscales apart. A non-Gaussian distribution was found using Kolmogorov-Smirnoff method. Thus, Spearman’s correlation coefficients were used to estimate intra and inter-rater reliability and to analyze associations between DSMP total and subscale scores and Mann–Whitney U test was used to compare children younger and older than 11 years old and those in different insulin regimens (Conventional versus Flexible). Wilcoxon test was used to compare DSMP scores between adolescents and their caregivers. The predictive validity between was HbA1c levels and DSMP scores was evaluated by Spearman’s correlation coefficient and multiple regression analysis. SPSS V20.0 package was used. A p value of ≤0.05 was considered statistically significant in all analyses.

## Results

The characteristics of the 102 patients (three were excluded due to low insulin dose use), DSMP total and each subscale scores are presented in Table 1.

**Table 1:** Patient characteristics, DSMP scores and Cronbach´s alpha

Patient characteristics		
Gender	Female = 54.9%	
Age (mean ± SD)	11.3 ± 3.2 years old	
Diabetes duration (mean ± SD)	6.2 ± 3.2 years	
Insulin regimen	58.8% Conventional/41.2% flexible	
Caregiver responder	Mother = 84%	
Family income (mean ± SD)	USD 15,682 ± 11,220	
HbA1c (median)	9.0% (8–11)	
HbA1c according to the age (median)	<11 years = 8.0% (8–9)/≥ 11 years = 10.0% (9–11)	
HbA1c according to insulin regimen (median)	Flexible = 8.0% (8-10)/Conventional = 10.0% (8–11)	

DSMP scores (maximum score)	(Median)	Cronbach's alpha
Total (77)	49.5 (39.0–57.0)	0.79
Subscales		
Exercise (12)	6.0 (3.0–0.0)	0.73
Hypoglycemia (10)	7.0 (6.0–9.0)	0.37
Diet (17)	9.0 (7.0–12.0)	0.47
Blood glucose testing (24)	18.0 (14.0–21.0)	0.67
Insulin (16)	9.0 (6.0–12.0)	0.44

Total score was significantly higher in patients younger than 11 years old (median: 54.0 vs 55.5; p < 0.001) as well as for all subscales, except that of diet. There was no difference between DSMP total and subscale scores between boys and girls. Patients in Flexible Regimen had better total scores than those in Conventional Regimen (median: 55.5 vs 46.5; p < 0.001) and significantly higher scores in exercise (8.0 vs 4.0; p = 0.041), diet (12.0 vs 8.0; p < 0.001) and blood glucose testing (19.0 vs 16.0; p < 0.001).

The DSMP total score demonstrated adequate internal consistency (Cronbach’s α 0.79), with a significant correlation with the subscale scores as follows: exercise (ρ = 0.67; p < 0.001), hypoglycemia (ρ = 0.56; p < 0.001), diet (ρ = 0.51; p < 0.001), blood glucose testing (ρ = 0.82; p < 0.001) and insulin (ρ = 0.71; p < 0.001).

In the inter-rater agreement analysis (n = 88) a moderate and significant correlation was demonstrate for the translated DSMP total scale (ρ = 0.55; p < 0.001) and all subscales (ρ = 0.65, 0.60, 0.58; p < 0.001) except for diet (ρ = 0.37; p = 0.004).

A moderate and significant correlation (ρ = 0.53; p < 0.001) was also demonstrated for the translated DSMP total scale as well as for diet (ρ = 0.52; p < 0.001) and blood glucose testing (ρ = 0.52; p < 0.001) subscales in test–retest reliability (n = 90).

DSMP total scores (ρ = −0.54; p < 0.001) and all subscales correlated significantly with HbA1c (exercise ρ = −0.41; p < 0.001, hypoglycemia ρ = −0.25; p = 0.01; diet ρ = −0.23; p = 0.02, blood glucose testing ρ = −0.51; p < 0.001, insulin ρ = −0.37; p < 0.001). When the association was tested in a multiple regression analysis, only blood glucose testing (p < 0.001) was correlated with HbA1c. DSMP total and subscale scores did not differ between adolescents and their caregivers when they were interviewed separately.

The hypoglycemia subscale revealed that no patients were using apparatuses for diabetes identification like necklaces, pendants, bracelets, and the glucose monitoring scale showed that no patient was using ketonemia/ketonuria strips.

## Discussion

Cronbach’s α coefficient values confirmed the reliability and internal validity of this DSMP translated version. The validation was also possible due to the application of the test–retest and inter-interviewer agreement that showed a moderate and significant correlation (>0.5). However, the inter-agreement coefficient was weaker than that demonstrated in the original validation (r = 0.94). A potential explanation for that was the subjectivity of an open interview and patients’ uncertainty about the frequency of their attitudes for each interviewer.

There was a moderate correlation between HbA1c and DSMP total score in our population. This result was the same for both English and Spanish DSMP versions applied in the Hispanic population of the United States [7].

Study participants presented higher HbA1c levels (9.5%) and a lower median total score (49.5) when compared to previous DSMP validation reports [4, 5, 8], except for Brazilian validation in adults [8]. The mean family income was equivalent to US$15,682 versus US$36,000 reported by Lewin [6]. Socio-demographic and cultural barriers have already been described in developing countries as factors related to a weak self-care behavior and poor glycemic control [12]. Age was an important predictor of good diabetes self-management in our study. Both the scores and HbA1c levels were progressively worse in older children [13]. Furthermore, diabetes duration was longer in our study population than in previous studies (6.2 × 4.9 [4] × 2.9 years [5]), which may be associated to a worse metabolic control due to a progressive beta-cell failure.

Patients on flexible regimen also showed better glycemic control and scores, possibly because patients with a better understanding of self-management are usually recommended a more intensive insulin therapy.

When answering the hypoglycemia subscale, many patients reported carrying only the ID card inside backpacks and wallets [4] due to shame of the disease within their social group. Although diabetes identification apparatuses are strongly recommended by ISPAD [14], our population generally avoids them.

The monitoring subscale disclosed lack of ketonemia/ketonuria testing during intercurrent illnesses probably because, in Brazil, ketonemia/ketonuria strips are not distributed by the public health system and the poor incentive to their use by the health team may have influenced such attitude.

The limitations of this study included a moderate test–retest reliability and inter-rater agreement, the lack of a “gold standard” comparison for self-management behavior, no patients on insulin pump therapy, and a population with poor metabolic control. Finally, the lack of use of apparatuses for diabetes identification and the absence of ketonemia/ketonuria strips supply could have interfered in the questionnaire internal consistency.

The translated DSMP version revealed that our population performs intensive monitoring, but is not yet able to change insulin doses according to each different situation (Table 1) [15]. For our diabetes team, adjusting insulin levels and ketosis monitoring during intercurrent diseases, as well as reinforcement of diabetes acceptance behavior will be the main points of our educational program from now on.

## Conclusion

DSMP-translated version into Brazilian Portuguese language was demonstrated to be a reliable and valid tool to assess DM1 self-management in youths.

## Abbreviations

DM1: type 1 diabetes
DSMP: diabetes self-management Profile
ID: identification
ρ: rho
